# Robots at the Chairside: A Narrative Review of Robotic-Assisted Surgery Revolutionizing Dental Implantology in the Digital Dentistry Era

**DOI:** 10.7759/cureus.102185

**Published:** 2026-01-24

**Authors:** Vaishnav Vinodkumar, Shankar S Menon, M Bharathkrishnan, Arun Kurumathur Vasudevan, Biju Balakrishnan, Maya Rajan Peter, Reshma Suresh

**Affiliations:** 1 Periodontics, Amrita School of Dentistry, Amrita Vishwa Vidyapeetham, Kochi, IND

**Keywords:** accuracy of implant placement, dentistry, oral implantology, oral surgery, robotic dental implant surgery, robotic implant, robotics, surgical robotics, yomi

## Abstract

The advent of robotic-assisted technologies has introduced a paradigm shift in dental implantology, aiming to enhance surgical precision, safety, and predictability. Robotic implant surgery integrates advanced imaging, computer-aided planning, and mechanized execution to overcome the inherent limitations of conventional freehand and static/dynamic navigation techniques. Traditional implant placement is heavily dependent on clinician experience, tactile feedback, and intraoperative judgment, which may result in deviations in implant positioning, prosthetic misalignment, or damage to vital structures. Robotic systems, such as Yomi (Neocis, Miami, FL, USA), Remebot (Beijing Baihui Weikang Technology Co., Ltd., Beijing, China), and Yakebot (Beijing Yakebot Technology Co., Ltd., Beijing, China), utilize haptic, visual, and combined feedback mechanisms to guide implant osteotomy and placement, thereby reducing human error, hand tremors, and operator fatigue.

Robotic platforms employ either semi-active or fully autonomous modalities, each offering distinct levels of surgical autonomy while maintaining surgeon oversight. Semi-active systems provide positional constraints and guidance, allowing surgeons to control drill motion within pre-planned trajectories, whereas active systems can execute osteotomies and implant insertion autonomously under real-time monitoring. These systems leverage three-dimensional imaging, cone-beam computed tomography (CBCT), optical markers, and force/torque sensors to achieve sub-millimetric accuracy in implant positioning. Furthermore, integration of artificial intelligence and machine learning algorithms enables adaptive responses to patient movement, variations in bone density, and thermal monitoring, simulating the tactile perception of experienced surgeons.

Beyond accuracy, robotic systems offer advantages in minimally invasive surgery, including reduced tissue trauma, improved flapless approaches, decreased surgical time in experienced hands, and enhanced ergonomics for clinicians. Moreover, robotic platforms hold potential for complex procedures such as full-arch rehabilitation and zygomatic implant placement, where conventional techniques pose greater risk. In conclusion, robotic-assisted dental implantology represents a transformative advancement, combining technological innovation with surgical precision to enhance clinical outcomes. Continued development and rigorous clinical evaluation are imperative to maximize its utility, broaden accessibility, and facilitate the transition from experimental to standard practice in contemporary implant dentistry.

## Introduction and background

Dental implant therapy is a predictable and widely accepted modality for the rehabilitation of partial and complete edentulism. Long-term implant success depends on accurate three-dimensional positioning in relation to available bone, adjacent anatomical structures, and the planned prosthetic restoration [1,2]. Deviations from the intended implant position are likely to compromise esthetics, biomechanical load distribution, and peri-implant tissue stability, all the while increasing the risk of surgical and prosthetic complications [3].

Conventional freehand implant placement is operator-dependent and influenced by clinical experience, tactile perception, and intraoperative judgment. Numerous studies have shown clinically relevant discrepancies between planned and achieved implant positions when freehand techniques are employed, particularly in anatomically complex sites [4]. To improve surgical precision, computer-aided implant surgery (CAIS) was introduced, including static surgical guides and dynamic navigation systems. While these approaches enhance accuracy when compared with freehand placement, they remain limited by guide instability, manufacturing errors, restricted intraoperative flexibility, and continued reliance on manual execution [5,6].

Robotic-assisted implant surgery is a further evolution of digital implantology, which integrates virtual planning with mechanized execution, using real-time feedback. Drawing on the advances in medical robotics, these systems aim to minimize human error by constraining or automating critical surgical steps while maintaining clinician supervision [7]. Unlike static or navigation-based systems, robotic platforms provide haptic, visual, or autonomous control of drill trajectory, depth, and angulation, thereby improving reproducibility and safety.

Robotics has been successfully implemented in multiple medical disciplines, including urology, neurosurgery, and cardiothoracic surgery, where precision and minimally invasive approaches are paramount [8]. Adoption in dentistry has historically been slower due to the confined operative field and the requirement for sub-millimetric accuracy. Recent developments in optical tracking, usage of force-feedback sensors, and real-time motion compensation have enabled the translation of robotic technologies into implant dentistry [9].

Dental implant robots may be classified according to their level of autonomy as passive (haptic-guided), semi-active, or active systems [10]. Currently available clinical platforms, such as the Yomi robotic system (Neocis, Miami, FL, USA), primarily operate as semi-active systems, which provide mechanical guidance while allowing surgeon-controlled execution [11]. These systems rely on cone-beam computed tomography (CBCT), digital impressions, and registration protocols to accurately translate virtual implant planning to the surgical field.

Robotic-assisted implant placement is particularly relevant in prosthetically driven workflows, immediate loading protocols, and complex rehabilitative scenarios, where cumulative errors associated with conventional techniques may compromise outcomes [12]. This narrative review aims to critically evaluate the current evidence available on robotic implant surgery, focusing on accuracy, clinical performance, limitations, and future directions relevant to implant practice.

## Review

Methodology

This manuscript was designed as a narrative review aimed at synthesizing and critically appraising the existing literature on robotic-assisted dental implant surgery. A narrative approach was intentionally selected because of the marked heterogeneity among currently available robotic systems, including differences in platform design, level of autonomy, surgical workflow, outcome definitions, and study methodologies. This variability limits meaningful quantitative pooling of data and precludes formal meta-analysis, thereby justifying a qualitative narrative synthesis of the evidence.

A comprehensive electronic literature search was conducted using PubMed/MEDLINE, Scopus, and Web of Science to identify relevant studies published up to December 2025. The search strategy incorporated a combination of free-text keywords related to robotic implant dentistry, including “robotic implant surgery,” “robot-assisted dental implants,” “dental surgical robotics,” “robotic dental surgery,” and “computer-assisted implant placement,” with Boolean operators applied as appropriate. Searches were restricted to articles published in the English language. In addition to database searches, the reference lists of included articles and relevant review papers were manually screened to identify additional studies that may not have been captured through electronic searching [13].

Studies were considered eligible for inclusion if they investigated robotic or robot-assisted systems used specifically for dental implant placement and if they were conducted as in vitro, animal, cadaveric, or human clinical investigations. Studies evaluating only static or dynamic computer-guided implant navigation without robotic assistance were excluded, as were articles addressing robotic applications in oral and maxillofacial surgery without specific relevance to dental implant placement. Editorials, narrative commentaries, conference abstracts lacking full-text availability, and non-peer-reviewed publications were also excluded.

The study selection process involved initial screening of titles and abstracts for relevance, followed by full-text assessment to confirm eligibility based on the predefined criteria. Selection was performed to ensure representative coverage of the spectrum of robotic systems and experimental models reported in the literature, rather than to achieve exhaustive inclusion.

Data were extracted descriptively from the included studies using a structured qualitative approach. Extracted information included the type and characteristics of the robotic system, the degree of robotic autonomy, the experimental or clinical setting, reported accuracy outcomes, surgical workflow characteristics, and feasibility or performance indicators. Given the narrative nature of this review and the heterogeneity of outcome measures and reporting formats, a quantitative data analysis was not performed. Instead, findings were synthesized using a qualitative narrative method, with emphasis placed on identifying consistent trends, technological capabilities, limitations, and emerging clinical implications of robotic-assisted implant placement.

The primary outcomes of interest were implant placement accuracy parameters, including linear deviation at the implant platform, linear deviation at the implant apex, and angular deviation between planned and placed implants. Secondary outcomes included feasibility of flapless implant placement, operative time, workflow efficiency, safety-related events, and early clinical outcomes when reported.

Formal risk-of-bias assessment tools were not applied, as this review was not designed to perform comparative quantitative analysis or graded evidence synthesis. Nevertheless, methodological limitations, study design constraints, and potential sources of bias were qualitatively considered during interpretation of findings and are discussed to provide balanced context for the reported outcomes [13].

Results

The reviewed literature demonstrated that robotic-assisted implant surgery consistently achieved high accuracy in implant placement. Reported linear deviations at the implant platform ranged between approximately 0.5 and 1.2 mm, while angular deviations were generally maintained below 4° [11,14]. These values were superior to those reported for freehand placement and comparable or improved relative to static and dynamic guided techniques.

Robotic systems also showed reduced variability in implant positioning across operators, suggesting little to no dependence on surgical experience [15]. Accuracy was maintained in anatomically challenging sites, including posterior regions and areas adjacent to critical anatomical structures [16].

Initial reports indicated longer setup times during early adoption phases; however, operative efficiency improved with experience, and learning curves appeared shorter when compared with dynamic navigation systems [17]. No major intraoperative complications directly attributable to robotic malfunction were reported. Built-in safety mechanisms, including force-feedback control and emergency override, contributed to procedural safety [10].

Early clinical data suggested favorable prosthetic outcomes, particularly in immediate loading and prosthetically driven cases, although long-term clinical data are limited [12].

Discussion

Robotic-assisted implant surgery represents a significant technological progression in contemporary implant dentistry, aiming to enhance surgical precision, procedural reproducibility, and safety. The present narrative synthesis indicates that robotic systems offer clinically meaningful advantages over conventional freehand and navigation-assisted approaches, particularly in complex, prosthetically driven, and minimally invasive treatment scenarios. These findings must be interpreted within the broader biological, biomechanical, educational, and ethical framework of implant therapy.

Clinical Relevance of Precision in Implant Placement

The accuracy of implant positioning is a fundamental determinant of treatment success, influencing prosthetic feasibility, biomechanical loading, and peri-implant tissue health. Minimally invasive implant placement strategies, particularly flapless surgery, require high positional fidelity due to the absence of direct visualization of underlying anatomical structures [18]. In this context, robotic-assisted implant surgery provides controlled execution of preoperative planning, enabling accurate osteotomy preparation while minimizing surgical uncertainty [18].

Preservation of peri-implant soft tissue architecture is closely linked to surgical precision. Flapless approaches supported by accurate guidance have been associated with reduced postoperative morbidity and maintenance of periosteal blood supply, which may contribute to favorable soft tissue healing [19]. Robotic systems, by constraining drill trajectory and depth, provide the precision required to safely execute such approaches [18,19].

Short-Term Clinical Outcomes and Peri-Implant Tissue Considerations

Although current evidence is predominantly short-term, early clinical reports suggest that robotic-assisted implant placement does not adversely affect peri-implant tissue health. Implant survival rates reported in short-term follow-up studies are comparable to those achieved with conventional techniques [20]. Furthermore, precise implant positioning may reduce prosthetic over-contouring and facilitate optimal emergence profile design, which are important determinants of long-term peri-implant health.

The prevention of peri-implant disease remains a major concern in implant dentistry. Improper implant positioning has been identified as a contributing factor to plaque accumulation and peri-implant inflammation [21]. By enhancing positional accuracy and prosthetically driven placement, robotic systems may indirectly reduce biological complications, although long-term evidence is required to substantiate this potential benefit [20,21].

Ethical and Regulatory Considerations in Robotic Surgery

As robotic systems become increasingly integrated into clinical workflows, ethical considerations surrounding autonomy, responsibility, and informed consent assume greater importance. Current dental robotic platforms function under the supervision of the clinician; nevertheless, the delegation of mechanical execution to robotic systems necessitates a clear delineation of professional responsibility [22]. Clinicians must retain full accountability for diagnosis, treatment planning, and intraoperative decision-making.

Patients should be adequately informed about the role of robotic assistance in their treatment, including potential benefits, limitations, and alternatives. Transparent communication is essential to maintaining patient trust as advanced technologies are introduced into routine clinical care [22].

Biomechanical Implications of Implant Positioning

From a biomechanical standpoint, accurate implant placement is essential for optimal load distribution and long-term stability. Implant angulation and depth influence stress patterns at the bone-implant interface, with mal-positioned implants demonstrating increased marginal bone strain and mechanical complications [23]. Robotic-assisted implant surgery may therefore contribute to improved biomechanical conditions conducive to long-term success, by reducing angular and linear deviations.

Accurate placement is particularly critical in full-arch rehabilitations and immediately loaded protocols, where cumulative positional errors can compromise prosthetic passivity and increase mechanical risk [24]. Robotic systems facilitate the precise execution of prosthetically driven planning, supporting biomechanically favorable implant distribution.

Bone Quality, Drilling Control, and Thermal Considerations

Bone density and drilling technique play a crucial role in primary implant stability and osseointegration. Excessive heat generation during osteotomy preparation may impair bone healing and jeopardize implant integration [25]. Robotic systems equipped with controlled drilling parameters and force-feedback mechanisms may reduce the risk of thermal trauma by maintaining consistent pressure and rotational speed.

Objective assessment of bone resistance during drilling may also enable adaptive modulation of surgical parameters, potentially optimizing primary stability across varying bone densities [26]. While these capabilities are promising, further clinical validation is required [25,26].

Operator Dependence, Standardization, and Educational Impact

Implant placement outcomes have traditionally been influenced by clinician experience and manual dexterity. Variability in surgical execution remains a challenge, particularly in academic and multi-operator environments. Robotic-assisted implant surgery has demonstrated the potential to reduce operator-dependent variability by constraining execution within predefined spatial parameters [27].

This standardization may benefit clinician training by allowing the supervised execution of implant placement with enhanced safety. However, excessive reliance on robotic assistance should not replace the development of foundational surgical skills. Balanced integration into educational curricula is essential to ensure that trainees develop both technological competence and sound clinical judgment [28].

Robotic Implant Systems Currently Available for Clinical Use

Several robotic-assisted implant systems have reached clinical application, each differing in design philosophy, level of autonomy, and method of surgeon-robot interaction. Among these, the Yomi system represents the most extensively reported dental robotic platform in the literature. Yomi is a surgeon-guided, haptic-feedback robotic system that provides real-time physical guidance during osteotomy preparation while allowing the clinician to retain control over drill activation and advancement. Clinical reports have demonstrated that Yomi-assisted implant placement achieves significantly reduced angular and linear deviations compared with freehand techniques, particularly in partially edentulous and anatomically constrained cases [11,14].

In contrast, Remebot (Beijing Baihui Weikang Technology Co., Ltd., Beijing, China) adopts a more automated robotic arm-based approach, wherein preplanned implant trajectories are executed through mechanically constrained drilling under continuous tracking. This system emphasizes reproducibility and standardization, particularly in full-arch and multiple implant rehabilitations, where cumulative placement errors may compromise prosthetic passivity. Early clinical experiences suggest that Remebot-assisted placement offers high positional fidelity comparable to navigation-based systems while reducing surgeon-dependent variability [16,29].

Emerging systems such as Yakebot (Beijing Yakebot Technology Co., Ltd., Beijing, China)/YakeBot-type platforms reflect ongoing efforts to integrate robotic control with digital implant workflows, including CBCT-based planning and real-time tracking. Although peer-reviewed clinical data on these platforms remain limited, their development underscores a broader industry trend toward semi-autonomous and workflow-integrated robotic implant placement. Collectively, these systems highlight the evolving spectrum of robotic assistance, ranging from haptically guided surgeon-controlled execution to increasingly automated mechanical constraint models [16].

Importantly, despite differences in system architecture, all currently available dental robotic platforms function as assistive technologies rather than autonomous surgical agents. Clinical responsibility for diagnosis, treatment planning, and intraoperative decision-making remains entirely with the clinician. The variability among platforms also underscores the importance of system-specific training, case selection, and understanding of technological limitations to ensure safe and effective clinical implementation [11,16].

Practical Limitations and Economic Considerations

Despite their advantages, robotic implant systems face substantial barriers to widespread adoption. High acquisition and maintenance costs remain significant limitations, particularly in private practice settings [28]. Additionally, system setup, calibration, and training requirements may initially increase procedural complexity.

Ergonomic constraints related to robotic hardware, including limited access in posterior regions and in patients with restricted mouth opening, necessitate careful case selection. Until these limitations are addressed through technological refinement, robotic assistance should be applied selectively where its benefits clearly outweigh logistical challenges [28].

Evidence Gaps and Need for Long-Term Data

A major limitation of the current literature is the lack of long-term clinical outcome data. Most available studies focus on placement accuracy and short-term feasibility, with limited reporting on marginal bone loss, peri-implant tissue stability, and patient-reported outcomes [29]. While improved accuracy is intuitively advantageous, its direct impact on long-term biological success must be confirmed through longitudinal randomized clinical trials.

Standardization of outcome reporting and consensus on clinically meaningful accuracy thresholds would further strengthen the evidence base and facilitate comparison across technologies [29].

Future Directions and Digital Integration

Future advancements in robotic-assisted implant surgery are expected to focus on enhanced system intelligence, seamless digital integration, improved ergonomics, and greater clinical accessibility. As digital dentistry continues to evolve, robotic platforms are likely to become increasingly interconnected with diagnostic imaging, virtual treatment planning, and prosthetic design workflows. Deeper integration with CBCT, intraoral scanning, and computer-aided design/computer-aided manufacturing systems may allow real-time synchronization between surgical execution and prosthetically driven planning, thereby reducing cumulative errors across the digital workflow and further enhancing precision [15,28].

The incorporation of artificial intelligence and machine learning algorithms represents a particularly promising direction in the evolution of dental robotics. Predictive analytics may enable real-time assessment of bone quality, drilling resistance, and primary stability, allowing adaptive modulation of surgical parameters during osteotomy preparation. Such developments could support personalized implant placement strategies based on patient-specific anatomical and biomechanical variables, moving robotic-assisted surgery beyond static execution toward responsive and data-driven intervention [22,28]. In the long term, artificial intelligence may also facilitate preoperative risk stratification, treatment outcome prediction, and optimization of implant positioning to minimize biological and mechanical complications [10,22].

Advances in haptic feedback, sensor technology, and force-control systems are expected to further refine surgeon-robot interaction. Improved tactile feedback may enhance intraoperative awareness of bone density transitions and cortical engagement, thereby supporting safer drilling in anatomically sensitive regions. Prior work on haptic systems in robot-assisted surgery highlights the importance of force-feedback in maintaining surgical control and preventing unintended tissue damage [9]. Concurrently, improvements in robotic arm dexterity and miniaturization of hardware could address current ergonomic limitations, particularly in posterior regions and in patients with restricted mouth opening, expanding the range of clinical indications suitable for robotic assistance [28].

From an educational and standardization perspective, robotic systems may play an increasingly important role in structured surgical training and competency assessment. Implant placement outcomes have traditionally shown variability related to operator experience and manual dexterity [17,27]. Robotic assistance has the potential to reduce this variability by constraining execution within predefined spatial parameters, thereby supporting safer supervised training environments and promoting reproducibility across operators and institutions [15,28]. Simulation-based training modules integrated with robotic platforms could further allow objective performance assessment while maintaining patient safety.

Economic considerations will remain central to the future adoption of robotic implant systems. High acquisition and maintenance costs currently limit widespread clinical implementation, particularly in private practice settings. However, continued technological maturation, increased competition, and broader adoption may contribute to cost reduction over time. Integration of robotics into comprehensive digital dental ecosystems may further enhance cost-effectiveness by streamlining workflows, reducing corrective interventions, and improving overall treatment predictability [28].

Despite rapid technological progress, the transition of robotic-assisted implant surgery from an emerging innovation to a routine clinical modality will depend on the availability of robust long-term clinical evidence. Most existing studies emphasize placement accuracy and short-term feasibility, with limited data on marginal bone stability, peri-implant tissue health, and patient-reported outcomes [20,21,29]. Well-designed randomized clinical trials, standardized outcome reporting, and longitudinal follow-up will be essential to determine whether the observed improvements in surgical precision translate into sustained biological and prosthetic benefits.

Future research should also aim to establish consensus on clinically meaningful accuracy thresholds and comparative effectiveness across different robotic and navigation systems. Such standardization would strengthen the evidence base, facilitate cross-study comparisons, and support evidence-based clinical decision-making [16,29]. Ultimately, ethical oversight, clinician-driven implementation, and patient-centered evaluation will be critical to ensuring that continued advances in robotic technology translate into meaningful improvements in implant dentistry [10,22].

## Conclusions

Robotic-assisted implant surgery represents a significant advancement in contemporary implant dentistry, reflecting the broader transition toward precision-based, digitally integrated clinical workflows. By combining detailed preoperative planning with real-time mechanically constrained or assisted execution, robotic systems address many limitations inherent in freehand and conventional computer-guided implant placement. The available evidence indicates that robotic assistance enhances placement accuracy, reduces operator-dependent variability, and improves the consistency with which planned implant positions are translated into clinical reality. Precise implant positioning facilitates restorative accuracy, supports immediate loading protocols, and may contribute to improved peri-implant tissue stability by enabling optimal emergence profile design and load distribution. Furthermore, robotic systems introduce advanced safety features, including force-feedback control and motion compensation, which are especially valuable in anatomically sensitive regions and procedures performed under local anesthesia. Despite these promising attributes, robotic implant surgery remains in an early phase of clinical adoption. High acquisition costs, technical complexity, ergonomic constraints, and limited long-term outcome data currently restrict widespread implementation. Moreover, while robotic systems may standardize surgical execution, they do not replace the need for sound clinical judgment, comprehensive diagnosis, and meticulous treatment planning. The clinician’s role remains central in case selection, decision-making, and management of biological and mechanical complications. In conclusion, robotic-assisted implant surgery has the potential to redefine standards of care by enhancing precision, predictability, and safety. With evidence-based adoption and appropriate clinical indications, robotic systems may become an integral component of advanced implant practice.
